# Mutations in Hsp90 Cochaperones Result in a Wide Variety of Human Disorders

**DOI:** 10.3389/fmolb.2021.787260

**Published:** 2021-12-08

**Authors:** Jill L. Johnson

**Affiliations:** Department of Biological Sciences and Center for Reproductive Biology, University of Idaho, Moscow, ID, United States

**Keywords:** CS domain, Aha1, tetratricopeptide repeat, FKBP, FNIP1, chaperonopathy

## Abstract

The Hsp90 molecular chaperone, along with a set of approximately 50 cochaperones, mediates the folding and activation of hundreds of cellular proteins in an ATP-dependent cycle. Cochaperones differ in how they interact with Hsp90 and their ability to modulate ATPase activity of Hsp90. Cochaperones often compete for the same binding site on Hsp90, and changes in levels of cochaperone expression that occur during neurodegeneration, cancer, or aging may result in altered Hsp90-cochaperone complexes and client activity. This review summarizes information about loss-of-function mutations of individual cochaperones and discusses the overall association of cochaperone alterations with a broad range of diseases. Cochaperone mutations result in ciliary or muscle defects, neurological development or degeneration disorders, and other disorders. In many cases, diseases were linked to defects in established cochaperone-client interactions. A better understanding of the functional consequences of defective cochaperones will provide new insights into their functions and may lead to specialized approaches to modulate Hsp90 functions and treat some of these human disorders.

## Introduction

Cytosolic Hsp90 is an abundant and essential molecular chaperone that mediates the folding of hundreds of cellular proteins, called clients. Hsp90 inhibition directly or indirectly impacts the function of up to 10–15% of all human proteins (Wu et al., 2012). Humans have two isoforms of cytosolic Hsp90: Hsp90α (encoded by HSP90AA1), and Hsp90β (encoded by HSP90AB1) (Chen et al., 2006). While both isoforms are abundantly expressed, there are some tissue-specific differences in expression (Dean and Johnson, 2021). In addition to Hsp90, client proteins interact with the molecular chaperone Hsp70 in an ordered cycle characterized by the presence of differing cochaperones. These cochaperones have a wide variety of functions, such as modulating Hsp90 ATPase activity or conformational changes or targeting clients to Hsp90. The most complete model of Hsp90 function is based on an ATP-dependent cycle of glucocorticoid receptor (GR) folding. Unfolded GR bound to Hsp70 progresses through a loading complex consisting of Hsp70, Hsp90 and Hop (encoded by STI1P) to a mature complex consisting of folded GR, Hsp90, and p23 (encoded by PTGES3). Additional cochaperones also interact with GR and influence its function (Panaretou et al., 2002; Pratt and Toft, 2003; Kirschke et al., 2014; Schopf et al., 2017). Altered cochaperone levels and/or complexes have dramatic effects on GR activity (Reynolds et al., 1999; Echeverria et al., 2016).

As the understanding of the range of Hsp90 functions has expanded (Gvozdenov et al., 2019), the number of identified Hsp90 cochaperones has also increased. There are roughly 50 human cochaperones that vary in client specificity, abundance, and tissue distribution (Taipale et al., 2014; Dean and Johnson, 2021). These cochaperones are roughly grouped by how they interact with Hsp90. One large group of contains a p23-like domain that shares homology with crystallin, termed a CS domain (Garcia-Ranea et al., 2002), which interacts with the amino terminus of Hsp90. Two of the main proteins in this group are p23 and Sgt1 (encoded by SUGT1). Although each of these cochaperones share a similar domain, they differ with regard for preference for the ATP-bound or nucleotide-free conformation of Hsp90 and client preference (Ali et al., 2006; Zhang et al., 2008).

Another large group, which contains Hop, contains tetratricopeptide repeat (TPR) or similar domains, and interacts with the carboxy-terminus of Hsp90 (Schopf et al., 2017). Although they share a common binding site, cochaperones in this group bind different Hsp90 conformations and have differing impacts on client function and Hsp90 ATPase activity (Prodromou et al., 1999; Riggs et al., 2004). A third set, which interacts primarily with the middle domain, does not contain either TPR or CS domains. This set includes Aha1, which strongly activates ATPase activity of Hsp90 (Panaretou et al., 2002). Some of the other cochaperones in this group regulate Hsp90, in part, by competing with Aha1 for Hsp90 interaction (Woodford et al., 2016). Studies in yeast suggest that a small set of cochaperones are part of the core Hsp90 machinery required for the folding of a wide range of clients, while others may have functions restricted to a few clients (Sahasrabudhe et al., 2017; Biebl et al., 2020). In humans, some cochaperones preferentially interact with one Hsp90 isoform or the other, which further contributes to the specificity of their effects (Chadli et al., 2008; Echeverria et al., 2016).

The functions of Hsp90 and cochaperones in cancer cells is well established and has been the subject of recent reviews (Calderwood and Gong, 2016; Vartholomaiou et al., 2016). Mutations in many cochaperones have been identified in tumor cells (https://www.cbioportal.org/). However, a detailed analysis of those changes is beyond the scope of this review. Changes in the expression of some of the cochaperones during aging or neurodegenerative disease has been noted (Brehme et al., 2014; Herold et al., 2016; Calderwood and Murshid, 2017; Lackie et al., 2017; Shelton et al., 2017; Bohush et al., 2019). In addition to those disorders listed above, altered cochaperone levels have been linked to inflammatory and heart diseases, asthma susceptibility, and dementia (Metcalfe et al., 2005; Sinclair et al., 2013; Patel et al., 2016; Bonham et al., 2018; Lee et al., 2019; Huang et al., 2020; Salhi et al., 2021). While most of the observed changes are at the transcriptional level, copy number variations in HSP90AB1 and some cochaperones have been identified (Metcalfe et al., 2005; Tomita-Mitchell et al., 2012; Zhang et al., 2019; Xu et al., 2021).

Mutations in genes encoding chaperones, most notably mitochondrial chaperonins Hsp60 and Hsp10, which are homologous to bacterial GroEL/GroES, and the small Hsp, Hsp27, have been previously found to cause a variety of pathologic conditions such as abnormalities in the nervous, muscular, or other tissues. These disorders have been termed chaperonopathies, and since most chaperones assist the folding of multiple proteins, the specific phenotypes of a chaperonopathy will depend on what function is impaired or abolished. (Macario et al., 2005; Macario et al., 2010; Cappello et al., 2013; Cappello et al., 2014; Lupo et al., 2016; Bulone et al., 2019; Comert et al., 2019). The goal of this review is to take advantage of recent advances and dissemination of genome sequencing technology to examine defects associated with mutation or deletion of specific human Hsp90 cochaperones. The results link chaperonopathies and altered Hsp90 cochaperone function. The variety of phenotypes demonstrates the unique functions of individual cochaperones and helps identify Hsp90-client-cochaperone interactions critical for human health.

## Disorders Linked to Mutations in Cochaperone Proteins

The reasons that cytosolic Hsp90 requires such large group of cochaperones is largely unknown. There is evidence that cochaperones exhibit client specificity, which suggests that the large number of cochaperones increases the Hsp90 clientele (Taipale et al., 2014). However, the folding pathway has been elucidated for only a small number of clients and it is largely unknown whether there are truly distinct folding pathways for diverse clients. As an alternate approach to identify unique functions of cochaperones, a literature review was used to identify whether disease-linked mutations have been found in any of the roughly 50 human cochaperones. Mutations in about 20 different cochaperones were found. Further analysis of the role of individual cochaperones in disease could help elucidate functions of both Hsp90 and cochaperones. In some cases, specific disorders were linked to mutations in single cochaperones, highlighting unique cochaperone functions. Since many cochaperones share common Hsp90 interacting domains, analysis of these interactions may be useful to understand the basis of selective cochaperone effects. As summarized in Table 1, the identified mutations result in a variety of phenotypes that affect heart, muscle, eye, and brain functions. For simplicity, cochaperones are identified by the gene name unless there is specific mention of the encoded protein.

**TABLE 1:** Hsp90 cochaperone-encoding genes with mutations linked to disease.

**Primary Ciliary dyskinesia**	**Neurodevelopment or neuromuscular disorders**
DYX1C1, PIH1D3, SPAG1 Taipale et al. (2003), Olcese et al. (2017), Paff et al. (2017), Wang et al. (2020b), Guo et al. (2020), Aprea et al. (2021)	STUB1 Shi et al. (2014), Hayer et al. (2017), Genis et al. (2018), Chen et al. (2020), Chiu et al. (2020), Mengel et al. (2021), Ravel et al. (2021)
	TOMM70 Dutta et al. (2020), Wei et al. (2020)
**Eye disease**	FKBP8 Tian et al. (2020)
AIPL1 Rashid et al. (2020), Sacristan-Reviriego et al. (2020), Xu et al. (2020)	CYB5R4 Suzuki et al. (2020)
UNC45B Hansen et al. (2014)	DNAJC7 Farhan et al. (2019), Wang et al. (2020a), Jih et al. (2020), He et al. (2021), Sun et al. (2021)
**Heart and muscle disorders**	FKBP6 Metcalfe et al. (2005)
UNC45B Dafsari et al. (2019), Donkervoort et al. (2020)	**Other**
PDCL3 Billon et al. (2020)	TSC1 Tuberous sclerosis Mowrey et al. (2020), Mastrangelo et al. (2021)
ITGB1BP2 Palumbo et al. (2009), Ruppert et al. (2013)	UNC45A Bone fragility Esteve et al. (2018)
FKBP6 Mescheriakova et al. (2019)	FKBP4 Androgen insensitivity Ilaslan et al. (2020)
FNIP1 Niehues et al. (2020)	AIP Pituitary Disease Bilbao Garay et al. (2020), Dal et al. (2020)
**Male infertility**	
FKBPL Male infertility Sengun et al. (2021)	
FKBP6, Zhang et al. (2007), Greither et al. (2020)	
**Inflammatory disease**	
SUGT1, Andreoletti et al. (2017)	

## Cilia Defects

A recent review of discussed the role of Hsp90 cochaperones in the assembly of dynein arm complexes (Fabczak and Osinka, 2019). Primary ciliary dyskinesia results from defective cilia and flagella beating and is characterized by airway disease, infertility, and laterality defects. Mutations in DYX1C1, PIH1D3 and SPAG1 result in primary ciliary dyskinesia. Most are deletion or truncation mutants that disrupt protein production (Taipale et al., 2003; Olcese et al., 2017; Paff et al., 2017; Guo et al., 2020; Wang et al., 2020b; Aprea et al., 2021). However, an inactivating missense mutation in PIH1D3 alters a conserved residue within the Hsp90-interacting CS domain (Olcese et al., 2017). Additional cochaperones with suspected links to cilia-related diseases are encoded by PIH1D1, PIH1D2, SUGT1, NUDC, NUDCD3, and RPAP3 ((Fabczak and Osinka, 2019) and (https://diseases.jensenlab.org/Search)) (Table 2). Studies with model organisms containing mutations or deletions of some of these cochaperones confirm the presence of ciliary defects (Chandrasekar et al., 2013; Tarkar et al., 2013). This indicates that a significant part of the Hsp90 machinery cooperates in the assembly of dynein complexes.

**TABLE 2:** Cochaperone-client associations linked to human disorders.

Client	Genes encoding cochaperones linked to client function
Glucocorticoid receptor	FKBP4, FKBP5, PTGES3, AHSA1, STIP1, USP19
Androgen receptor	FKBP4, FKBPL, SGTA
Aryl hydrocarbon receptor	AIP
Dynein arms	PIH1D1, PIH1D2, SUGT1, NUDC, DYX1C1, PIH1D3, SPAG1, NUDCD3, RPAP3
Myosin	UNC45B, UNC45A
Actin	PDCL3
Integrins	ITGB1BP2
TDP-43	STIP1, DNAJC7
NOD1	SUGT1
mTOR	FNIP1, FNIP2, TSC1, FKBP8
PDE6	AIPL1
PDE4A5	AIP
Bcl-2	FKBP8

## Leber Congenital Amaurosis

AIPL1 (encoded by AIPL1) cooperates with Hsp70 and Hsp90 to facilitate folding and assembly of retinal cGMP phosphodiesterase (PDE6) (Hidalgo-de-Quintana et al., 2008; Sacristan-Reviriego and van der Spuy, 2018). PDE6 is a known Hsp90 client, and prolonged treatment with Hsp90 inhibitors results in reduced PDE6 levels (Aguila et al., 2014). Mutations in AIPL1 are associated with Leber congenital amaurosis, a severe form of inherited retinal degeneration (Yadav et al., 2017; Rashid et al., 2020; Sacristan-Reviriego et al., 2020; Xu et al., 2020). Mice lacking AIPL1 serve as a disease model of Leber congenital amaurosis. Retinas from AIPL1 knockout mice exhibit rapid degeneration of both rods and cones and destabilization of cGMP phosphodiesterase (Ramamurthy et al., 2004; Singh et al., 2014). Multiple missense mutations in AIPL1 have been analyzed to determine their effect on Hsp90 interaction and/or PDE6 functions (Sacristan-Reviriego et al., 2020). Mutations that altered either the FKBP domain or the TPR domain of AIPL1 resulted in reduced Hsp90 interaction and reduced PDE6 activity in cells coexpressing AIPL1 and PDE6. Thus, both the FKBP and TPR domains are critical for AIPL1 function.

## Neurodevelopmental and Neurodegeneration Disorders

CHIP (encoded by STUB1) is a ubiquitin ligase that plays a key role in regulating targeting misfolded Hsp90 clients for degradation (Connell et al., 2001). Mutations in STUB1 have been found in patients with both recessive and dominant forms of ataxia, which is characterized by a lack of muscle control or coordination of voluntary movements (Ravel et al., 2021). Some other mutations cause ataxia plus additional severe phenotypes (Shi et al., 2014; Hayer et al., 2017). Mice lacking the gene exhibit defects in motor and sensory function, plus additional defects (Shi et al., 2014). The disease correlates with overall loss of CHIP function, and missense mutations in the TPR domain or the ubiquitin-ligase domain are known to affect localization and/or activity (Shi et al., 2014; Hayer et al., 2017; Genis et al., 2018; Chen et al., 2020; Chiu et al., 2020; Mengel et al., 2021; Pakdaman et al., 2021).

TOMM70 encodes a protein that is part of the mitochondrial preprotein import machinery (Young et al., 2003). Missense mutations in TOMM70 cause ataxia combined with white matter abnormalities and other defects. Both of those mutations exhibited loss of function in an animal model (Dutta et al., 2020). Additional TOMM70 variants have been linked to reduced overall mitochondrial and OXPHOS deficiencies, resulting in severe anemia, lactic acidosis, and developmental delay (Wei et al., 2020). Some of the mutations alter residues in the TPR domains, although direct analysis of their impact on Hsp90 interaction is unknown.

Mutations in a different TPR-containing cochaperone, FKBP38 (encoded by FKBP8), have been identified as risk factors for spina bifida, a neural tube defect. FKBP38 localizes to the mitochondria and helps regulate apoptosis through Bcl-2 interaction (Shirane and Nakayama, 2003). Mice deficient in FKBP8 exhibited neural tube defects due to unrestrained apoptosis (Shirane-Kitsuji and Nakayama, 2014). Each of the mutants identified resulted in an increase in cellular apoptosis and/or altered FKBP38 levels. The structure of FKBP38 bound to the TPR acceptor site is known (Blundell et al., 2017), and two of the mutants identified altered conserved residues in the TPR domain (Tian et al., 2020).

DNAJC7 encodes TPR2, a protein that contains TPR domains in addition to a J domain, a domain involved in Hsp70 interaction (Brychzy et al., 2003). Mutations in DNAJC7 have been linked to amyotrophic lateral sclerosis, a progressive nervous system disease that affects nerve cells in the brain and spinal cord. Most of observed mutations were protein-truncating variants, and in some cases protein loss was confirmed (Farhan et al., 2019; Jih et al., 2020; He et al., 2021). However, one missense mutation that alters a residue in one of the TPR domains was found (Wang et al., 2020a). A specific role for TPR2 in pathogenic events has not yet been identified, but yeast models suggest a role for Hsp90 and cochaperones in TDP-43 misfolding, a hallmark of the disease (Lin et al., 2021).

NADH cytochrome b5 oxidoreductase (Ncb5or) protects beta-cells against oxidative stress and lipotoxicity (Kalman et al., 2013). In mice, loss of Ncb5or has been linked to insulin-deficient diabetes and other deficiencies (Xie et al., 2004; Stroh et al., 2016; Stroh et al., 2018) Ncb5or contains a CS domain that is shared with other Hsp90 cochaperones, but its functional interaction with Hsp90 has not been characterized (Benson et al., 2019). A mutation in CYB5R4 that leads to a truncated version of Ncb5or has been implicated in neurodevelopmental disorders (Suzuki et al., 2020), but a potential mechanistic defect is unknown.

## Heart and Muscle Disorders

The basic mechanism in muscle involves the interaction of the protein filaments myosin and actin, both of which are affected by mutations in Hsp90 cochaperones. The TPR-containing cochaperones encoded by UNC45A and UNC45B promote myosin folding (Lee et al., 2014; Lehtimaki et al., 2017; Bujalowski et al., 2018). Mutations that alter the myosin-binding domain of UNC45B were found in a patient with congenital muscle disorder (Dafsari et al., 2019). Additional mutations were associated with progressive myopathy (Donkervoort et al., 2020). Yet another mutation in UNC45B resulted in cataracts, a result supported by studies in an animal model (Hansen et al., 2014). Mutations in UNC45A, which encodes GCUNC45, a related cochaperone, were associated with a range of symptoms, including impaired hearing and bone fragility (Esteve et al., 2018).

PhLP2A (encoded by PDCL3) is an Hsp90 interacting protein that does not contain either TPR or CS domain that is involved in the folding of actin (Krzemien-Ojak et al., 2017). Loss of function alleles of PDCL3 were identified in patients with megacystis-microcolon-intestinal-hypoperistalsis syndrome, which affects muscles in the bladder and intestines (Billon et al., 2020). Integrins are transmembrane links between extracellular contacts and the actin microfilaments. Melusin (encoded by ITGB1BP2) is a muscle-specific integrin-interacting protein that contain a CS domain and has important cardioprotective functions (Brancaccio et al., 2003; Sbroggio et al., 2008; Tarone and Brancaccio, 2015). Mutations in ITGB1BP2 have been detected in families of patients affected by hypertension or cardiomyopathy (Palumbo et al., 2009; Ruppert et al., 2013).

Loss of folliculin interacting protein 1 (encoded by FNIP1) due to inactivating mutations results in immunodeficiency and heart defects due to disruption of essential metabolic regulators AMPK and mTOR (Saettini et al., 2021). Hsp90 inhibition is known to target multiple components of mTOR signaling (Giulino-Roth et al., 2017). One of the FNIP1 mutations associated with disease is a predicted missense mutation in a domain known to be involved in regulation of Hsp90 interaction and FNIP stability (Sager et al., 2019; Niehues et al., 2020), suggesting that this alteration affects Hsp90-related functions. Two additional cochaperones, encoded by FNIP2 and TSC1, are also involved in mTOR signaling, and the three all cochaperones function to inhibit Hsp90 (Woodford et al., 2016; Woodford et al., 2017; Sager et al., 2018; Sager et al., 2019). Mutation of TSC1 is associated with tuberous sclerosis complex, an autosomal dominant multisystem disorder that affects the brain, skin, heart, kidneys, and lung (Mallela and Kumar, 2021). Most of the mutations disrupt protein production, but a few examples of missense mutations in the region implicated in Hsp90 interaction have been identified (Bykhovskaya et al., 2017; Woodford et al., 2017).

## Mutations in FK506-Binding Proteins have Been Linked to Various Disorders

One set of cochaperones, including the ones encoded by FKBP8 and AIPL1, which were described above, contains a FK506-binding domain in addition to TPR domains (Annett et al., 2020). A mutation that alters a residue in the TPR domain of FKBP6 was identified in patients with multiple sclerosis, but it has not been conclusively linked to disease (Mescheriakova et al., 2019). Other genetic variations in FKBP6 have been linked to the neurodevelopmental disorder Williams-Beuren syndrome (Metcalfe et al., 2005), congenital heart malformations (Tomita-Mitchell et al., 2012) and male infertility (Zhang et al., 2007; Greither et al., 2020). Loss of function alleles of FKBPL have also been associated with male infertility (Sengun et al., 2021), and a mutation in FKBP4 has been associated with androgen insensitivity (Ilaslan et al., 2020).

Aryl hydrocarbon receptor interacting protein AIP is similar to AIPL1. Mutations in the AIP gene are the most frequent genetic cause for familial isolated pituitary adenomas, which predispose individuals to acromegaly and gigantism (Bilbao Garay et al., 2020). Mutations that disrupted the ability of AIP to interact with clients, such as the aryl hydrocarbon receptor and phosphodiesterase 4A5 were linked to disease (Morgan et al., 2012).

## Link Between Cochaperones and Inflammatory Disease

Inflammatory bowel disease is an umbrella term for a group of illnesses that includes Crohn’s disease, ulcerative colitis, and other diseases. Mutations that affect the NOD signaling pathway are a major contributor to the disease. The Sgt1 cochaperone (encoded by SUGT1) is essential for NOD1 activation (da Silva Correia et al., 2007). SUGT1 mutations have been identified but not yet definitely linked to disease. One of those is located in a TPR domain (Andreoletti et al., 2017). Variation in the cochaperones encoded by PTGES3L and FKBPL has also been potentially linked to various inflammatory disease (Hocevar et al., 2018; Bianchi et al., 2021), but more analysis is required. Single-nucleotide variants in the HSP90AB1 and HSP90B1 genes have been proposed to modulate chronic obstructive pulmonary disease (Ambrocio-Ortiz et al., 2021), and Hsp90 is known to regulate inflammatory processes (Nizami et al., 2021). More research is needed to understand the role of Hsp90 and cochaperones in inflammatory disease.

## Mutations in Cochaperones for the Hsp90 Isoform in the Endoplasmic Reticulum have Also Been Associated With Disease

While the main focus of this review is on cytosolic Hsp90, higher eukaryotes express Hsp90 isoforms in both the mitochondria (TRAP1) and the ER (GRP94) (Chen et al., 2006; Yoshida et al., 2013). Both TRAP1 and GRP94 have important functions and there is interest in developing isoform specific inhibitors (Seo, 2015; Sanchez-Martin et al., 2020). These isoforms of Hsp90 do not require a large number of cochaperones. However, there are a few examples of cochaperones that play critical roles in client maturation. GRP94 is required for folding of immunoglobulin light chains, Toll-like receptors and other proteins. A few different cochaperones for GRP94 have been identified, including OS-9 (Christianson et al., 2008), MZB1 (Rosenbaum et al., 2014), and CNPY3 (Liu et al., 2010). Mutations in CNPY3 are associated with epileptic encephalopathy (Mutoh et al., 2018), demonstrating that cochaperones for other Hsp90 isoforms also have critical functions relevant to human disease.

## Hsp90-Cochaperone-Client Interactions Relevant to Human Disorders

**FIGURE 1:**
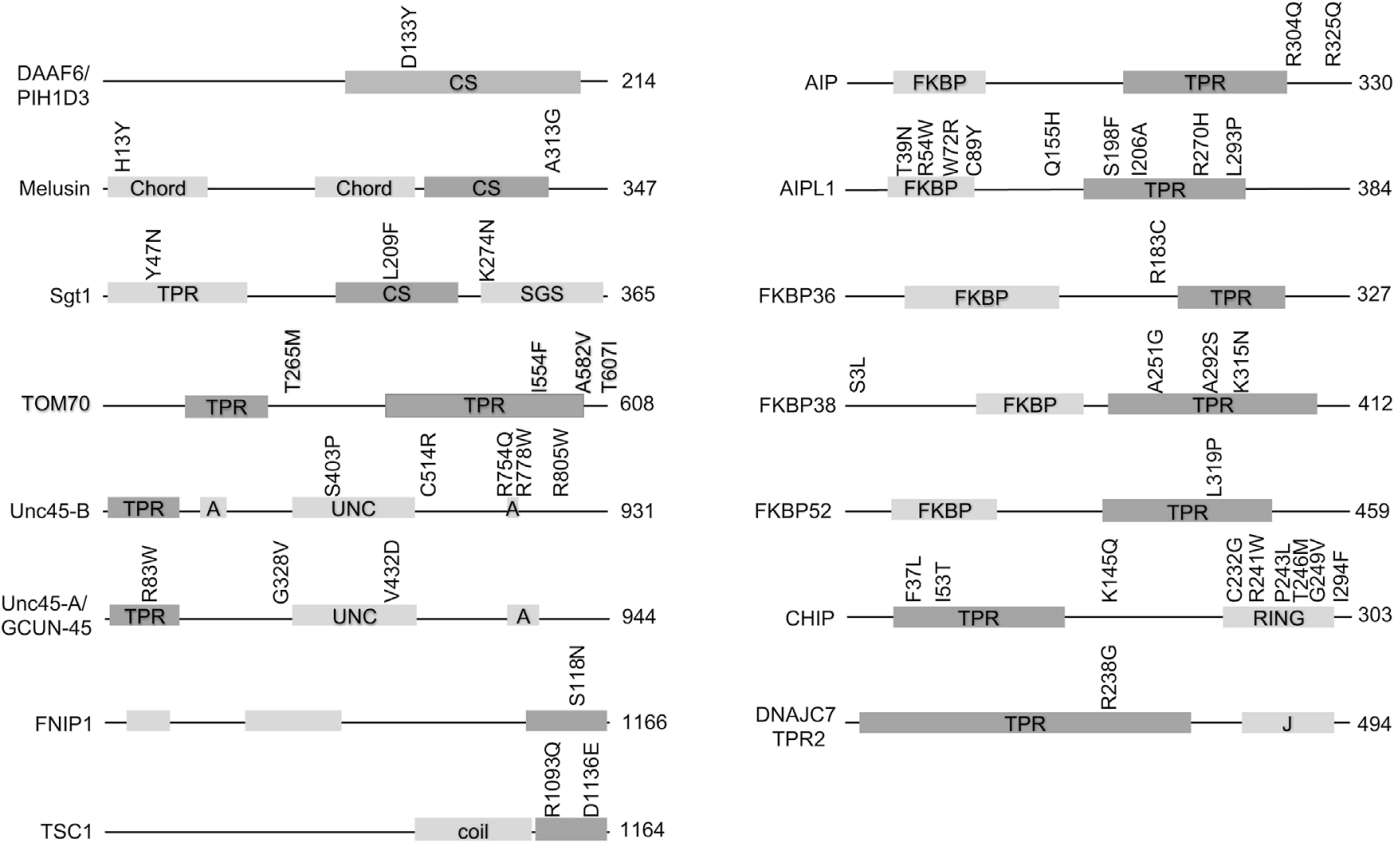
Missense mutations in cochaperones linked to human disease. Schematic of cochaperone protein domains and the location of disease-associated mutations. The size of each protein (in amino acids is shown). The domain known or predicted to mediate Hsp90 interaction is shaded in the darker color. Abbreviations: CS= (Chord and Sgt1 like), Chord (Cysteine- and histidine-rich domains), TPR (tetratricopeptide repeat), SGS (Sgt1-specific), A (armadillo), UNC (uncoordinated, myosin-interacting), coil (coiled-coil), FKBP (FK506-binding protein), RING (RING-finger U-box). J = DnaJ-like domain).

Hsp90 cochaperones are a diverse group of proteins that, in addition to the Hsp90 interacting domain, usually contain other domains with known functions. Figure 1 shows the domain structure of cochaperones with missense mutations implicated in disease. In many cases, missense mutations are located within the Hsp90-interacting domain, implying that the defective Hsp90-cochaperone interaction results in disease. However, it should be noted that many of these mutations are newly identified, and more studies are needed to determine whether the mutations alter Hsp90 interaction. It is also possible that the mutations affect Hsp90-independent functions of the cochaperones. Further, some of these cochaperones, including but not limited to TOM70, DNAJC7 and HOP (Scheufler et al., 2000; Young et al., 2003; Timsit and Negishi, 2014; Assimon et al., 2015), interact with both Hsp90 and Hsp70 and thus it is not possible to assign the defects to Hsp90 interaction without further analysis. Finally, it is also possible that the mutations affect the ability of cochaperones to be post-translationally modified. As summarized in recent reviews, there is growing evidence that post-translational modifications on chaperones and cochaperones regulate their interactions and function (Backe et al., 2020; Nitika et al., 2020).

Identification of Hsp90-client-cochaperone interactions important for human health may be helpful to develop assays to test for Hsp90 inhibitor selectivity or potential negative side effects. A list of Hsp90 clients known or suspected to be affected by mutation of the cochaperones listed above are summarized in Table 2. In some cases, additional cochaperones are also known to interact with some of those listed. For example, the GR is one of the most widely studied Hsp90 clients, and changes in activity of GR and/or other clients due to altered abundance of associated cochaperones has been linked to mood disorders, autism, anxiety, psychotic illness, depression, and altered pain susceptibility (Sinclair et al., 2013; Patel et al., 2016; Baker et al., 2018; Lee et al., 2019; Lou et al., 2021; Mokha et al., 2021; Salhi et al., 2021; Szczepankiewicz et al., 2021). Similarly, androgen receptor activity is highly dependent on cochaperones encoded by FKBP4, FKBPL, and SGTA (Paul et al., 2014; Ilaslan et al., 2020; Sengun et al., 2021).

## Cochaperones Without Identified Human Mutations

Deletion of some mammalian cochaperones results in inviability, while loss of others has mild effects. Mice lacking HSP90AA1 survive to adulthood, but deletion of HSP90AB1, PTGES3 or STIP1 caused embryonic lethality (Grad et al., 2006; Grad et al., 2010; Beraldo et al., 2013). Deletion of SGTA resulted in reduced viability and growth defects (Philp et al., 2016), while deletion of PPP5 or FNIP2 in mice causes mild effects (Amable et al., 2011; Hasumi et al., 2015). In contrast, deletion of USP19 protected mice against muscle wasting in response to glucocorticoids (Bedard et al., 2015). The Genome Aggregation Database (https://gnomad.broadinstitute.org/) is a publicly available database on human genetic variation. Based on the number of protein truncating variants observed in the dataset, each gene has a pLI score that reflects the likely tolerance of gene loss. A score of 0 suggests that loss of the gene may be tolerated, while a score of 1 suggests that it is not tolerated (Karczewski et al., 2020). Although there are known pitfalls of relying too heavily on pLI scores (Ziegler et al., 2019), this provides a useful comparison of the likely effect of cochaperone mutation. As shown in Table 3, available data suggests that deletion of some cochaperones is not tolerated. This includes genes encoding cochaperones like Hop, p23 and Cdc37 that are required for activity of a wide range of Hsp90 clients and likely have essential functions. The yeast homologs of those cochaperones are known to be required for activity of multiple clients (Sahasrabudhe et al., 2017; Schopf et al., 2017; Verba and Agard, 2017). In other cases, there may be functional redundancy with other cochaperones or other cellular pathways that masks the effect of cochaperone mutation. Alternatively, the encoded cochaperone may interact with either a limited set of clients or only nonessential clients.

**TABLE 3:** Hsp90 cochaperones without known genomic mutations linked to disease. pLI (probably of loss interference) numbers are from https://gnomad.broadinstitute.org/.

Cochaperone	pLI= >0.85–1 (1 = likely lethal)	Intermediate	pLI ∼ 0–0.1 (tolerated)
CS domain	PTGES3, USP19, CACYBP, CHORDC1	NUDCD3	NUDCD1, NUDCD2, PIH1D1, PIH1D2, HACD3
TPR domain	STIP1, PPP5C, SGTA, NASP	FKBP5	PPID, TTC4, RPAP3, TOMM34, TTC1, CRNKL1
Other	CDC37, CDC37L1, AHSA1, FNIP2		

## Conclusion

This review is an attempt to consolidate available information about known genetic variation in genes encoding human Hsp90 cochaperones. One conclusion is that there are many similarities between previously characterized chaperonopathies and disorders linked to Hsp90 cochaperone mutations. This includes ciliary defects, cataracts and other eye diseases, neuropathies or neurodegenerative diseases, and various myopathies (Macario et al., 2005). More research is needed to understand these complex disease associations and determine the extent of functional overlap between different types of chaperones. Another conclusion is that loss of different cochaperones causes distinct effects. This is consistent with prior studies that indicate that even cochaperones that share homologous Hsp90-interacting domains exhibit client specificity (Riggs et al., 2004; Taipale et al., 2014). Another conclusion is that while some processes, such as maturation of the glucocorticoid receptor or the assembly of dynein arms, require groups of cochaperones that may be able to compensate for each other, in other cases, such as with AIPL1, cochaperone function may be limited to a very small number of critical clients. A better understanding of the specific role of AIPL1 in PDE6 function may provide important information about why closely related cochaperones, such as AIP, are apparently unable to compensate for the loss of AIPL1.

Due to the importance of Hsp90 in supporting cancerous growth, development of Hsp90 inhibitors remains a priority as a tool to treat cancer. The general Hsp90 inhibitors that bind the ATP-binding site are associated with unwanted side effects. Alternative strategies include development of Hsp90 isoform specific inhibitors (Khandelwal et al., 2018; Mak et al., 2019; Mishra et al., 2021) or compounds that block functions of specific cochaperones (Guy et al., 2015; Stiegler et al., 2017; Wang et al., 2017; Singh et al., 2020; Serwetnyk and Blagg, 2021). A greater understanding of the possible consequences of cochaperone inhibition is necessary to assess the potential negative side effects of some of these more selective inhibitors of Hsp90 function.
